# Values as Predictors of Religious Experience in the Lives of Seminary Students of Philosophy and Students of Physics

**DOI:** 10.1007/s10943-016-0235-6

**Published:** 2016-04-12

**Authors:** Stanisław Głaz

**Affiliations:** Jesuit Academy Ignatianum, Ul. Kopernika 26, 31-501 Kraków, Poland

**Keywords:** Terminal values, Seminary students of philosophy, Students of physics, Religious experience

## Abstract

The aim of this study was to show the preferences of terminal values of personal and social character and the level of religious experience: God’s presence and God’s absence, as well as to examine the relationship between the two variables in the groups of seminary students of philosophy and students of physics. The following methods were applied in the study: Rokeach Value Survey and Głaz’s Scale of Religious Experience. The study was conducted amongst university students in Kraków (Poland). The results of 100 correctly completed sets of questionnaires were analysed. The results analysis proves that seminary students of philosophy have a higher level of religious experience: God’s presence and God’s absence than students of physics. Seminary students of philosophy most preferred terminal values in personal and in social character. In the group of seminary students of philosophy, from amongst the four most preferred terminal values, two have a significant relation with the experience of God’s presence and God’s absence, whereas in the group of students of physics only one of them has a significant relation with the experience of God’s absence.

## Introduction

Research studies show that values have significant relations with the religious experience and perform an important regulatory function in the process of development of a human being’s religious life (Halama and Halamová 2005; Popielski 2008; Głaz 2014b; Krok 2014, 2015b). It is suggested that there exists a significant relationship between terminal values of personal and social character and religious experience: God’s presence and God’s absence. The aim of this article is to compare the relation between these two components in the lives of seminary students of philosophy and students of physics.

### Values

The concept of values in social sciences has more than one interpretation. In the field of psychology, two different standpoints concerning the understanding of values have developed. The first one is the subjective standpoint, which refers values to the subjective sphere, meaning it assumes that “it is people who create values”. Something becomes a value because it is preferred, desired, considered to be a value (Brzozowski 2003). The other standpoint, in turn, is represented by objectivists (Reykowski, Rokeach, Schwartz). According to them, values exist regardless of the subject. The values lie in the subject and are a feature of the entity. Something is a value itself, and therefore, a person should learn about values and realise them. According to Reykowski (1978), a value is what manifests its cognitive representation. He talks about a values network. The values network’s components include cognitive elements arranged in terms of the value attributed to them. More recently, Schwartz conceptualised values as desirable, trans-situational goals, varying in importance and serving as guiding principles in people’s lives. Values, being socially approved verbal representations of basic motivations, play an important role in our functioning and are linked to motivational goals. Schwartz (1999) distinguished 10 distinct motivational goals that are expressed as the following types of values: power, achievement, hedonism, stimulation, self-direction, universalism, benevolence, tradition, conformity, and security (Schwartz 2014). The source of values is deeply ingrained in individuals’ minds, which enables them to discover standards and modes of conduct. Specific understanding of the values proposed Rokeach (1973). According to that scientist, though, values are a set of concepts or convictions. Convictions are part of personality and religiosity. Personality, in turn, is a system of convictions composed of a variety of subsystems. The most important part of all subsystems is the structure of one’s own “I”, followed by values. An individual value, in Rokeach’s view, is a constant conviction that a person’s specific mode of conduct or the end-state of human existence is personally and socially superior to the opposite or converse mode of behaviour or the ultimate state of existence. Therefore, Rokeach classifies values referring to the highest goals of human existence (such as personal freedom, salvation) as *terminalvalues*, which have a personal or pro-social character, and values connected with general modes of human behaviour (ambitious, responsible) called *instrumentalvalues*, which have a moral and competence dimension. According to Rokeach, values have a beyond situational character and provide standards for the choice of behaviour as well as for the assessment of one’s own behaviour and that of other people. Rokeach believes that all people, regardless of time and place, respect the same values, although to a different extent. The system in which particular terminal and instrumental values function is characterised by a specific organisation (Rokeach 1969). The internalisation of values is favourable for the individual’s conscience control of their own behaviour and is a mean for achieving autonomy in their development. The result of this is the possibility of pointing to most preferred values by individuals themselves. In the present paper, in order to define the hierarchy of values amongst academic youth, the Rokeach Value Survey was applied in the form adapted to the Polish conditions by Brzozowski (1986).

### Religious Experience

Experience, in the broad meaning of the word, is a fundamental form of human cognition, and it denotes a certain process, “movement” of consciousness thanks to which a human being establishes contact with the reality with a view to getting to know it (Argyle 2000; Wilkinson 2010). This characteristic also refers to experience religious in character (Król 1999; Hood et al. 2009).

Some researchers describe religious experience as profoundly spiritual. It is perceived as a type of genuine and immediate contact with a power recognised as divine presence (Gruehn 1956) or divine reality (James 1908; Jung 1982), and it is a direct capture of the presence of religious (transcendental) reality (Biela and Tobacyk 1987; Walesa 2005). According to Otto (1968), religious experience is a specific feeling of holiness (*sacrum*). *Sacrum* influences a human being in two ways—it fills them with awe and fear (*mysteriumtremendum*), and simultaneously attracts and encompasses them (*mysteriumfascinans*). On the one hand, *sacrum* arouses fear in a person, anxiety, sometimes fright, and, on the other hand, it is appealing and arouses fascination. Religious experience is often accompanied by tension and one’s inner conflicts. It is because it is ambivalent in itself. A human being, when establishing contact with sacrum, experiences an ambiguous feeling: he sees his imperfection and sinfulness, and, at the same time, he desires meeting which is sacred. Obviously, these are by no means purely emotional states due to the fact that they engage the whole personality. Other scientists show that in origin it has no religious reference. For instance, it is thought that religious experience may be sourced by psychedelic drugs (Smith 1964; Clark 1989; Grof 1985; Głaz 2013a) or that religious experience could be a result of conflict between id and ego in the human being (Freud 1961a). According to Freud, religious experience is the “oceanic feeling”. By religious feeling, he means the simple and direct fact of a feeling of “the eternal” (which may very well not be eternal, but simply without perceptible limits, and as if oceanic). This feeling is in truth subjective in nature. Religious experience is also quite common to identify experiences invoked by both psychedelic substances (boundary experiences) or the brain activity stimulated by means of external tools (neurotheology) with religious experience caused by God’s doing (Newberg 2010). On the one hand, religious experiences provide new contents about man and the Absolute (Jung 1982), which favours the process of getting to know oneself (Frankl 1987) and the surrounding world better (Fromm 1966), but on the other hand, as claimed by Freud (1951, 1961b) and others (Ancona 1966), it falsely assures of God’s careful providence, which eventually leads to self-deception and neurotic disorders.

The analysis of the works of great Christian mystics such as Juan de la Cruz or Teresa de Avila shows that religious experience has an existential and dialogical character (Głaz 1998). It is more than simple experiencing of religious feelings; above all, it is present in a personal meeting of a human being and God. Gadamer (1986) seems right claiming that religious experience Christian mystices is a “meeting” and it should not be considered as a sole activity of the subject but as an event (*Ereignis*), meaning something that happens to man, such as a meeting with another person. Religious experience is a process taking place in the context of a specific religious tradition. Therefore, it is considered as part of two dimensions of human life: the community dimension and individual dimension (van Kaam 1964). It is also conditioned by the history of an individual and his or her society. According to other researchers, religious experience changes peoples’ way of perception of the world and their attitude to it (Allport 1950). It has an influence on shaping a person’s attitude to oneself, others, and God (Głaz 1998).

Experience of this kind would seem to share a specific structure, comprising the following elements: the object of religion (God personified), the subject of religion (a human being), and a religious relation personal in character (Głaz 1998). What decides about the kind of religious experience are elements contributed by both man and God, taking into consideration their uniqueness and independence. Man is often accompanied by a feeling of God’s presence and another time God’s absence. A human being has two completely separate kinds of religious experience: experience of God’s presence and God’s absence in life. These kinds of experience are something specific, practically unique. They often arouse contradictory feelings in a person. A human being’s experiencing of God’s presence is real, and this kind of experience often leaves a person with a feeling of happiness, content, even elation. Experiencing God’s absence, His lack in one’s own life, is accompanied by other kinds of feelings. Typically feelings of sadness, anxiety, and even a conviction of being rejected by God tend to dominate. The intensity of these feelings varies (Głaz 1996, 2014a). A human being, when entering a relation with God, acquires new contents through which God Himself “speaks” about His intentions towards him or her and the world (Głaz 2000). The human being responds to them with faith, love, and engagement (Głaz 2006b). In response to these features, the author of this paper, referring to Christian understanding of religious experience (Saint Jean de la Croix 1915; Tauler 1826)—created, on the basis of that concept, a tool for measuring experiences with a religious character: God’s presence and God’s absence. The scale will be employed for the purposes of the article. It consists of statements referring to the Christian religion which take in into account all of the dimensions described above: the cognitive, emotional, and behavioural experiences of God’s presence and God‘s absence.

### Problem and Objective Research

Two kinds of lifestyle are encountered in our culture: clerical and secular (lay). The clerical lifestyle is directed at self-realisation with respect to God and another person, whereas the secular one is directed at self-realisation with regard to another person and the surroundings. The former suggests that a human being’s care about their own development, experiencing happiness, and solitude takes place in an institutional religious group where a person finds fulfilment individually and socially. The development includes following Christ’s footsteps and rejecting certain values (marriage—in the Roman-Catholic Church) (Thoppil 2004). The latter—secular—suggests that a human being as a person finds fulfilment individually and socially when caring about one’s own development, gaining life happiness, being free, while living in certain community.

When choosing lifestyle and a university faculty, a person is guided by various criteria. One of them is the content of the curriculum for a given faculty. Another criterion is, for instance, being there for others and with others. It encompasses any kind of service manifesting itself in engaging for the sake of others. There are also more practical criteria, namely prospects of a good job or being popular in a given social circle. Motivations of this kind of choice can to a greater or lesser extent contribute to realisation of a person’s talents, choosing the right values, and to integrate development of his or her personality and religious life (Rulla et al. 1977; Uchnast 1983; Klinkosz and Iskra 2008, 2010; Krok 2015a).

According to some researchers, each lifestyle is accompanied by a certain level of religious experience (Rulla et al. 1977; Plante et al. 1996; Głaz 2013b) and a specific hierarchy of values (Brzozowski 2003; Popielski 2008). The studies conducted amongst seminarians, who belong to the Roman-Catholic Church, show that seminary students most often sought help for two reasons (Soiński 2001). Firstly, in order to get to know themselves—in this group the majority of seminary students were of the opinion that prayer served this purpose; the second reason was religious crisis and a decline of values, in which case they tended to seek the spiritual leader help. Soiński’s other research findings (2008) show that diocesan clerical students experienced a crisis of values more often than monastic clerical students preparing for ordination. Amongst the study subjects, a bigger group of secular students than clerical students claim that their attitude to values had changed when at university (Soiński 2009). Seminary students respected more values related to the Christian vocation, such as following in Christ’s footsteps, salvation, love, and the least respected were a comfortable life and pleasure; secular students, however, valued most personal freedom and true friendship. In turn, research by Zarzycka and Maslowski (2008) reveal that seminary students with a low level of dissimulation are characterised by more mature love and a greater openness to others than those with a high level of dissimulation. Głaz’s research findings (2006a) show that seminary students of philosophy have a higher level of experience of God’s presence than secular students of theology as well as a higher level of experience of God’s absence than female students of pedagogy. Other research by Głaz (2006b) reveals that in a group of male students of theology out of terminal values the strongest relation with experience of God’s presence has salvation, whereas in a group of female students mature love. Moreover (Głaz 2011), seminary students and students of sciences prioritise the same values (wisdom, pleasure, and family security).

According to Rokeach’s (1969) theories, terminal values are arranged within an organised hierarchical system and are a relatively permanent structure in an individual’s personality and religiosity. Terminal values personal in character focus strongly on an individual, whereas values of a pro-social dimension refer more to interpersonal relations and relate to the surroundings. What decides about the choice of values and their realisation is, on the one hand, a person’s sensitivity to values and, on the other hand, their attractiveness. A coherent system of values and their internalisation enriched by authentic religiosity increase the possibility of the occurence of coherent pro-social behaviours, which manifests itself in interpersonal relations, and also they define its aims and co-create the style of social relations and the forms of interpersonal communication, which has been proven by various research findings (Czerniawska 2000, 2002; Roccas 2005). Rokeach (1969) claims that a value most preferred by a person has a central position in his or her life and at the same time performs a significant regulatory function.

The problem of values and religious experience: God’s presence and God’s absence—aspects connected with self-realisation as a person—is a popular research area (Głaz 2013c, 2014b). However, this issue poses a challenge for scientists and calls for detailed studies. Empirical facts relating to values and religious experience: God’s presence and God’s absence found in the literature are rather ambiguous due to the fact that they depend on the specificity of the subject groups and the interpretation of a given problem adopted in the study procedure.

This paper focuses on an attempt to show the relation between terminal values and religious experience: God’s presence and God’s absence in the group of seminary students of philosophy, who live in a religious community, prepare for “being with others”, live their lives in accordance with evangelical advice, and follow the Christ’s footsteps, and in the group of students of physics, who are characterised by being open to external experience, belief in a rational world order, and fascination with material reality. In this context the following questions arise: To what extent does lifestyle—as a way of realisation of students’ individual traits—define and modify the corresponding world of terminal values, which perform an important regulatory function in their lives, as well as religious experience: God’s presence and God’s absence? To what extent do the most preferred values—as personal standards—imply religious experience: God’s presence and God’s absence of seminary students of philosophy and student of physics? On the basis of the presented theoretical material and earlier studies, the following hypotheses were formulated:

#### **Hypotheses**

1. There is a difference in the level of religious experience: God’s presence and God’s absence, and in the hierarchy of terminal values between seminary students of philosophy and students of physics.
2. The most preferred terminal values contribute considerably to the occurence of religious experience: God’s presence and God’s absence both in the group of seminary students of philosophy and in the group of students of physics.

## Methods

### Participants and Procedure

In order to obtain empirical material with the help of which the research problem can be solved, a study was conducted in Kraków in 2012 amongst university students. The group of students of physics consisted of young people at a state university (Jagiellonian University). The group of seminary students, though, consisted of clerics at a private university (Academy Ignatianum). All participants were Polish born. All the students declared belonging to the Roman-Catholic Church and have had religious experience of God’s presence and God’s absence. The age of the participants ranged between 21 and 27 (*M* = 23.8; *SD* = 2.02). The study was scheduled for after academic classes. All the participants had been personally instructed how to complete the questionnaires. Over a dozen of sets of questionnaires were only partly completed, and consequently, they were excluded from the analysis. The results of 100 correctly completed sets of questionnaires (50 by seminary students of philosophy and 50 by students of physics) were then analysed.

### Measures

In order to solve the research problem, the following instruments were applied:

#### The Scale of Religious Experience (SRE) by Głaz

The scale has three sub-scales. The first one describes the intensity of the experience of God in general (OB); Cronbach’s alpha coefficient of internal consistency is .92. The second scale serves to measure the intensity of the experience of God’s presence (OB), with Cronbach’s alpha coefficient of internal consistency at .94. The third scale describes the intensity of the experience of God’s absence (NB). Cronbach’s alpha coefficient of internal consistency is also high and stands at .91 (Głaz 2011). In the present study, the second and third sub-scales were used. Criterion validity: correlation (*p* Spearman) between the Scale of Religious Experience and Prężyna’s Scale of Religious Attitude is .63.

#### Rokeach Value Survey (RVS)

It consists of two sub-scales. Each of them distinguishes 18 values. One of the sub-scales, which was applied in the study, serves to establish the preferences of terminal values of personal and social character. Rokeach (1973) analysed the consistency of each value (test reliability) with the test–retest method (*N* = 250) and obtained for terminal values coefficients ranging from .51 to .88. The scale was adapted to the Polish conditions by Brzozowski (1986). The rank correlation coefficient between the Polish and American versions of the sub-scale of terminal values is .99.

### Statistical Analysis

The analysis of variance (ANOVA) was applied. The results analysis was carried out on the basis of mean values (*M*) and standard deviations (*SD*). In order to define a statistically significant relation between terminal values of personal and social character, and religious experience: God’s presence and God’s absence, the analysis of multiple stepwise regression was applied. Therefore, a decision was made to extract independent variables in order to see which and to what extent explain the variance of religious experience: God’s presence and His absence within the respective groups of the studied subjects.

## Results

The analysis of the obtained results allowed the verification of the hypotheses concerning the relation between terminal values in personal and in social character and religious experience of God’s presence and God’s absence in the lives of seminary students of philosophy and students of physics.

### The Level of Religious Experience of God’s Presence and God’s Absence in the Lives of Seminary Students of Philosophy and Students of Physics (Table 1)

The results obtained in the Scale of Religious Experience (SRE) (Table 1) indicate two differences significant in terms of religious experiences; namely, seminary students of philosophy have a higher level of religious experience of God’s presence (OB) (*F* = 21.01; *p* < 0.001) and a higher level of experience of God’s absence (NB) than males studying physics (*F* = 5.11; *p* < 0.01).

**Table 1:** Analysis of variance (ANOVA), value of the *F* test, and the level of significance *p* for the variables in the groups of seminary students of philosophy and students of physics obtained in the Scale of Religious Experience (SRE)

Variables	Seminary students of philosophy		Students of physics		*F*	*df*	*p*
	*M*	*SD*	*M*	*SD*			
OB	5.12	0.93	3.61	1.02	21.01	1	<0.001
NB	4.67	0.89	3.32	0.93	5.11	1	<0.01

### Ranking Distribution of Terminal Values of Personal and Social Character for the Groups of Seminary Students of Philosophy and Students of Physics (Table 2)

For the interpretation of the preferences of terminal values, the top four values with the highest ranks were considered. The results obtained in the Rokeach Value Survey (RVS) (Table 2) concerning terminal values show that seminary students of philosophy respect most values like inner harmony (4), wisdom (5), salvation (5), and freedom (6) (all in personal character), whereas male students of physics prefer terminal values such as a world at peace (5), pleasure (6), wisdom (7), and family security (7) (two in social character and two in personal character).

**Table 2:** Ranking distribution of terminal values in personal (p) and in social (s) character in groups of seminary students of philosophy and students of physics

Groups of students	Most preferred terminal values	Ranks
Seminary students of philosophy	Inner harmony (p)	4
	Wisdom (p)	5
	Salvation (p)	5
	Freedom (p)	6
Students of physics	A world at peace (s)	5
	Pleasure (p)	6
	Wisdom (p)	7
	Family security (s)	7

p—terminal value in personal character

s—terminal value in social character

### Relation Terminal Values with Experience of God’s Presence in the Groups of Seminary Students of Philosophy and Students of Physics (Table 3)

Five different terminal values have a significant relation with religious experience of God’s presence (OB) (Table 3). In the group of seminary students of philosophy these include: true friendship, salvation, wisdom, and social recognition. The first variable, namely true friendship, explains 11 % of the variance of experience of God’s presence (OB) (*R* = 0.33), and together all the values explain 38 % of the variance of that experience (OB) (*R* = 0.61). The goodness of fit of the obtained stepwise regression equation defines the test value *F*(4,45) = 5.49; *p* < 0.01. In the group of males studying physics, one terminal value counts—equality, which explains 12 % of the variance of the experience of God’s presence (OB) (*R* = 0.34). The goodness of fit of the obtained stepwise regression equation defines the test value *F*(1,48) = 4.22; *p* < 0.01.

**Table 3:** Independent variables relating to terminal values in personal (p) and in social (s) character explaining the variance of religious experience: God’s presence (OB)

Groups of students	Values explaining the variance of God’s presence (OB)	*B*	*R*	Percentage of explained variance (*R* ^2^ × 100 %)
Seminary students of philosophy	True friendship (s)	0.12	0.33	11
	Salvation (p)^a^	0.17	0.41	17
	Wisdom (p)^a^	0.22	0.55	30
Students of physics	Social recognition (p)	0.31	0.61	38
	Equality (s)	0.13	0.34	12

The results of the multiple stepwise analysis for seminary students of philosophy and students of physics. The results of the multiple stepwise regression analysis

^a^One of the four highest ranked values for the relevant group of students

### Relation Terminal Values with Experience of God’s Absence in the Groups of Seminary Students of Philosophy and Students of Physics (Table 4)

Five different terminal values have a significant relation with experience of God’s absence (NB) (Table 4). In the group of seminary students of philosophy, terminal values such as mature love, salvation, and freedom turned out to be significant variables. The first variable, that is mature love, explains 10 % of the variance of experience of God’s absence (NB) (*R* = 0.32), and all of the terminal values, in turn, explain 39 % of the variance of that experience (NB) (*R* = 0.62). The goodness of fit of the obtained stepwise regression equation defines the test value *F*(3,46) = 5.69; *p* < 0.01. In the group of students of physics, two terminal values count: an exciting life and wisdom. The first value—an exciting life—explains 20 % of the variance of experience of God’s absence (NB) (*R* = 0.45), whereas both of them explain 22 % of the variance of experience of God’s absence (NB) (*R* = 0.47). The goodness of fit of the obtained stepwise regression equation defines the test value *F*(2,47) = 4.02; *p* < 0.05.

**Table 4:** Independent variables relating to terminal values in personal (p) and in social (s) character explaining the variance of religious experience: God’s absence (NB)

Groups of students	Values explaining the variance of God’s absence (NB)	*B*	*R*	Percentage of explained variance (*R* ^2^ × 100 %)
Seminary students of philosophy	Mature love (s)	0.21	0.32	10
	Salvation (p)^a^	0.07	0.45	20
	Freedom (p)^a^	0.18	0.62	39
Students of physics	An exciting life (p)	0.12	0.45	20
	Wisdom (p)^a^	0.13	0.47	22

The results of the multiple stepwise regression analysis for seminary students of philosophy and students of physics. The results of the multiple stepwise regression analysis

^a^One of the four highest ranked values for the relevant group of students

## Discussion of the Results and Conclusion

This paper presents the author’s attempt to show the relations between terminal values in personal and in social character and religious experience: God’s presence and God’s absence in the lives of seminary students of philosophy and students of physics. For the interpretation of the preferences of terminal values, the top four values with the highest ranks were taken into consideration for the analysis. The verification of the study hypotheses and the analysis of the study problem are as follows.

The first part of the hypothesis, which suggests that there is a difference in the level of religious experience: God’s presence and God’s absence between seminary students of philosophy and students of physics, was fully confirmed. Seminary students of philosophy are accompanied by a higher level of experience of God’s presence and His absence than students of physics. According to the literature and some researchers’ suggestions (Głaz 2006b; Soiński 2008), this result was to be expected. The second part of hypothesis, which indicates that there is a difference in the hierarchy of terminal values between the group of seminary students of philosophy and the group of students of physics, was almost fully confirmed. The two groups of students prefer different terminal values. Out of the four most preferred terminal values, which define an individual’s most important goals and aspirations as personal standards, seminary students of philosophy pointed to four values in personal character (inner harmony, wisdom, salvation, and freedom), whereas students of physics chose two values in personal character (pleasure and wisdom) and two values of social character (a world at peace and family security). Only one value—wisdom—out of the four most preferred values was pointed to by both seminary students of philosophy and students of physics.

The hypothesis which suggests that the most preferred terminal values significantly contribute to the occurence of religious experience of God’s presence and God’s absence was confirmed only to a certain extent in the group of seminary students of philosophy as well as in the group of students of physics. In the group of seminary students of philosophy out of the four most preferred terminal values in personal character, salvation and wisdom are significantly related to experience of God’s presence, and, as far as religious experience of God’s absence is concerned, so are the terminal values—salvation and personal freedom. These values are in personal character. However, in the group of students of physics none of the four most preferred by them values has a significant relation with experience of God’s presence, and only one value respected by them, in personal character—wisdom—is significantly related to experience of God’s absence. In this case, Rokeach’s theory was confirmed only to a certain extent.

According to Rokeach’s (1973) theory, values create a hierarchical system of an individual’s convictions, aspirations, and goals, and their central role proves their important regulatory function in a human being’s personality and religious life. In this case, the analysis of the obtained results shows that of the eighteen terminal values it is rather values in personal character, which focus on a person or refer to an individual’s success, and not values of social dimension, that perform an important regulatory function both in the lives of seminary students of philosophy and in the lives of students of physics. It may imply that the current educational system and social life enhance independence; however, they fail to teach co-operation (team work) and do not respect gaining knowledge.

It was expected, in accordance with the vocation and lifestyle of seminary students preparing for priesthood, that seminary students of philosophy are accompanied by a higher level of experience of God’s presence and His absence than students of physics. Some research findings (Zarzycka and Maslowski 2008; Soiński 2008) indicate an existence of such a relation, and the present analysis confirmed its existence. A high result obtained in the Scale of Religious Experience suggests that the lifestyle in the religious community of seminary students of philosophy in the religious community favours self-realisation as a person and their vocation. Seminary students of philosophy treat seriously their preparation for completing the mission which they will be entrusted with by the religious community. They have more personal references to God, and they care more about their spiritual development than students of physics.

A greater number of terminal values were expected to have a significant contribution to the occurence of religious experience: God’s presence and His absence in the group of students of physics, which was not confirmed. It would suggest that students of physics reveal lack of internalisation of terminal values. According to Rokeach’s (1973) theory, this would suggest a lack of a coherent personality. People with incoherent personalities are not very successful in action, and their activity is often paralysed with inner conflicts they are unaware of.

In the group of seminary students of philosophy, the contribution of terminal values in the process of explaining the variance of experience of God’s presence ranges from 11 to 38 % and God’s absence from 10 to 39 %. In the group of students of physics, though, the contribution of terminal values in the process of explaining the variance of experience of God’s presence is 12 % and God’s absence from 20 to 22 %. It suggests that in this case terminal values, which define the students’ aspirations and goals and present a certain system of reference which allows young people to assess reality and direct their lives, a few times more strongly imply and strengthen experience of God’s presence and His absence in the lives of seminary students of philosophy than in the lives of students of physics.

Religious experience of God’s absence—and even more God’s presence—as an important element of young people’s religiosity, which enhance getting to know more oneself and the surroundings, is strongly conditioned by the regulatory function performed by terminal values in personal character, which means the ones which refer to university students’ process of personal development; a considerably less important role is played by terminal values of pro-social dimension, meaning the ones which demark correct interpersonal relations.

The analysis of the problem indicates that there exists a significant correlation between terminal values of personal and social character and religious experience: God’s presence and God’s absence. In spite of their correlations, each of these dimensions is autonomous in terms of its cognitive, behavioural, emotional, and evaluative aspects. Each of these decides about self-realisation of a person and about the way of living one’s own life. The choice of a lifestyle and the related university faculty creates conditions for gaining knowledge, and the realised system of values determines the cognitive network of seminary students of philosophy and students of physics; it activates and outlines standards of their behaviour, as well as performs an important function of the criteria determining their line of thinking and acting. Religious experience: God’s presence and God’s absence encourages students to deeper reflection and examination of their reality and the surroundings.

A current problem is emerging, namely that of academic youth in Poland departing from the dimension of institutional religiosity in favour of the aspects of religious experiences (Zarzycka and Masłowski 2008; Soiński 2006), which indicates a necessity to support this kind of studies. The studies I have embarked on constitute an essential contribution to solving this problem. Religious experience: God’s presence and God’s absence is one of the important elements of human religiosity. The need to identify factors supporting or deterring this kind of experience. A particularly important aspect of such studies is learning about the predictors of religious experience amongst academic youth. This study was conducted within the terms oft he Christian religion, in a country where the majority of inhabitants are Roman Catholic. Nevertheless, it may also serve as a model to prompt others to undertake further research of this kind—for example, amongst other Christian denominations.
